# The sacroiliac dysfunction and pain is associated with history of lower extremity sport related injuries

**DOI:** 10.1186/s13102-023-00648-w

**Published:** 2023-03-20

**Authors:** Sajjad Abdollahi, Rahman Sheikhhoseini, Mohammad Rahimi, Wendy E. Huddleston

**Affiliations:** 1 Department of Corrective Exercise & Sport Injury, Faculty of Physical Education and Sport Sciences, Allameh Tabataba’i University, Western Azadi Sport Complex Boulevard, Hakim Highway, Tehran, Iran; 2 Department of Corrective Exercise & Sport Injuries, Faculty of Sport Sciences, Shahid Rajaee Teacher Training University, Western Azadi Sport Complex Boulevard, Hakim Highway, Tehran, Iran; 3 Department of Rehabilitation Sciences & Technology, College of Health Sciences, University of Wisconsin-Milwaukee, Milwaukee, WI USA

**Keywords:** Injury risk, Musculoskeletal injuries, Prevalence, Sacroiliac Joint, Sports Injuries

## Abstract

**Background:**

The purpose of this study was to examine the association of sacroiliac joint (SIJ) dysfunction and pain with overuse and acute lower limb and pelvic girdle injuries of Iranian basketball players.

**Methods:**

In this cross-sectional study, basketball-related injury data were collected during 2019–2020 from 204 basketball players of the Iranian league using the online Information Retrospective Injury Questionnaire. A researcher then performed ten clinical tests to assess SIJ dysfunction and pain (five tests for dysfunction and five tests for pain). Data analysis was performed by logistic regression at the confidence interval of 95%.

**Results:**

Within our sample (n = 204), injury rates were calculated across sub-groups of athletes that had only SIJ pain (n = 19), only SIJ dysfunction (n = 67), both SIJ pain and dysfunction (n = 15) or no SIJ complaints (n = 103). Across these groups, a total of 464 injuries were reported. SIJ pain group reported 80 injuries (17.2%), SIJ dysfunction group reported 210 injuries (45.2%), both SIJ pain and dysfunction group reported 58 injuries (12.5%, and the no SIJ pain or SIJ dysfunction group reported 116 injuries (25.0%). Participants with SIJ pain were more likely to report previous pelvic girdle injuries (overuse: odds ratio (OR): 0.017; 95% CI: 0.005–0.56; p < 0.001 and acute: OR: 0.197; 95%CI: 0.101–0.384; p < 0.001) and also lower limb injuries (overuse: OR: 0.179, 95%CI: 0.082–0.392, p < 0.001). Participants with SIJ dysfunction only were likely to report acute pelvic girdle injuries (OR: 0.165; 95%CI: 0.070–0.387; p < 0.001) and acute lower limb injuries (OR: 0.165; 95%CI: 0.030–0.184; p < 0.001).

**Conclusion:**

The presence of SIJ dysfunction and pain is associated with a history of acute and overuse injuries in the pelvic girdle and lower limb. Thus, SIJ dysfunction and pain should be specifically evaluated and addressed when designing rehabilitation programs for sports-related injuries.

**Supplementary Information:**

The online version contains supplementary material available at 10.1186/s13102-023-00648-w.

## Background

Injuries and dysfunctions of the sacroiliac joint (SIJ) are critical to lower extremity function as this joint has been shown to play a vital role in the biomechanical and functional movements of athletes [1, 2]. In basketball, SIJ disorders are commonly associated with unilateral and repetitive biomechanical forces, such as jumping, landing, throwing, and single-leg stance. The SIJ provides the link for ground reaction forces between the lower extremities and trunk during weight-bearing activities [3, 4]. Thus, it allows load transfer from the lumbar spine to the lower extremities, and vice versa, depending on proper activation of muscles including abdominal, leg, and back musculature. Correct muscle activation allows for normal load transmission across the lumbopelvic region [5, 6]. When the sacroiliac joint (SIJ) doesn’t move properly because of the joint or surrounding soft tissue damage or lack of forced closure due to improper muscle activations and forces [7, 8], it is considered a disorder. Eight to 24% of athletes report SIJ disorders at some time [8–12]. However, the exact prevalence of SIJ pain or dysfunction among athletes remains unknown and is likely underdiagnosed because SIJ pain referral patterns often imitate low back pain (LBP) [8, 13], and 39% of patients with SIJ dysfunction have concomitant spinal pathology [13].

Additionally, susceptibility to lower extremity injury appears to increase in the presence of SIJ dysfunction [14]. To explain this relationship, anatomical studies have illustrated that an asymmetry in load transmission through the lumbopelvic region can lead to compensatory muscle activation that stabilizes the pelvis [15]. The delayed onset of activity of these proximal muscles in the supporting limb suggests a disruption of the normal rhythm of SIJ forced closure. This disruption can lead to a disturbance of load transference through the SIJ [16]. According to the compressive loading model proposed by Snijders et al. [3], athletes with SIJ dysfunction/pain demonstrate different loading patterns in the SIJ that may result in different neuromuscular activation patterns. The injury-related neurophysiological changes may affect the muscle activation pattern that occurs while weight loading transfers across the SIJ [3]. Thus, inadequate pelvic joint stability can be associated with specific disorders of the pelvic girdle, including lumbopelvic pain [17], groin [18], and lower extremity injuries [19, 20]. Furthermore, according to kinesiopathological models, athletes who must perform repetitive or asymmetrical loading (jumping, landing, tossing, and single-leg stance) may be more susceptible to musculoskeletal disorders like SIJ dysfunction [21, 22]. These repetitive movements may lead to cumulative microtrauma injuries in the SIJ that may be result in SIJ/low back pain disorders [23].

While a small body of literature exists regarding SIJ pathology (SIJ pain and SIJ dysfunction) on athletes and the association among SIJ disorders, pelvic girdle injuries, and lower limb injuries, further study is necessary to more fully comprehend the relationship of SIJ disorders with other injuries. The first goal of this study was to determine the prevalence of SIJ problems among Iranian basketball players. The second aim was to examine the association of SIJ problems (including dysfunction, pain or both) with a history of overuse and acute injuries of lower limb and pelvic girdle in Iranian basketball players. We hypothesized that a significant association would exist between SIJ problems (including dysfunction, pain or both) with a history of overuse and acute injuries of lower limb and pelvic girdle.

## Materials and methods

### Participants

In this cross-sectional study, participants were professional basketball players of the Iran league. A total of 204 male junior players agreed to participate in the present study. This sample of convenience was recruited by the first author who attended team camps and asked athletes to participate in the current study. The only inclusion criteria was that the player had to have played in the league the previous year (2019-20), regardless of current injury status or participation in rehabilitation at the time of the study. All participants were informed of the risks and benefits of study participation and were asked to sign an informed consent form before participating. The Research Ethics Committee of the Allameh Tabataba’i University approved all research processes and methods (IR.ATU.REC.1399.015). The authors confirm that all research was performed in accordance with relevant guidelines/regulations. Also, informed consent for publication of identifying images was obtained for online open-access publication.

### Data collection

#### Retrospective injury questionnaire

All survey and physical examination data were collected during the last 2 weeks of the 2020–2021 basketball season. A self-administered Retrospective Injury Questionnaire (RIQ) [24, 25], which was developed based on the standard questionnaire of sports injury registration [26], was used to collect data. The survey asks participants to reflect on injury occurrence over a preceding 12-month period. All subjects filled the questionnaire prior to any physical examination. The reliability of the Persian version of RIQ has been established previously [25]. RIQ contains information on personal characteristics, participation in sports activities, and the history of sports injuries. The athletes were asked to evaluate, as precisely as possible, the number of weekly training sessions, hours spent per session, and the number of games played during the previous season. The questionnaire contains questions on injury incidence over the last 12 months (including games and training). For each injury that happened over the previous year, athletes noted the anatomic location, type of injury, the nature of injury (i.e., acute or overuse), background (contact or non-contact, training, or competition), date of occurrence, and recovery time in the RIQ. An injury was considered reportable if it happened during participation in training sessions or matches, required medical attention by team physicians or medical staff, and led to at least one calendar day of lost training or activity restriction [27]. Acute injury was defined as injury caused by a distinct trauma with a specific identifiable event that caused the injury. Overuse injury was defined as being caused by a gradual onset without a single identifiable event that caused the injury [28].

### Physical exam procedures

**Fig. 1:**
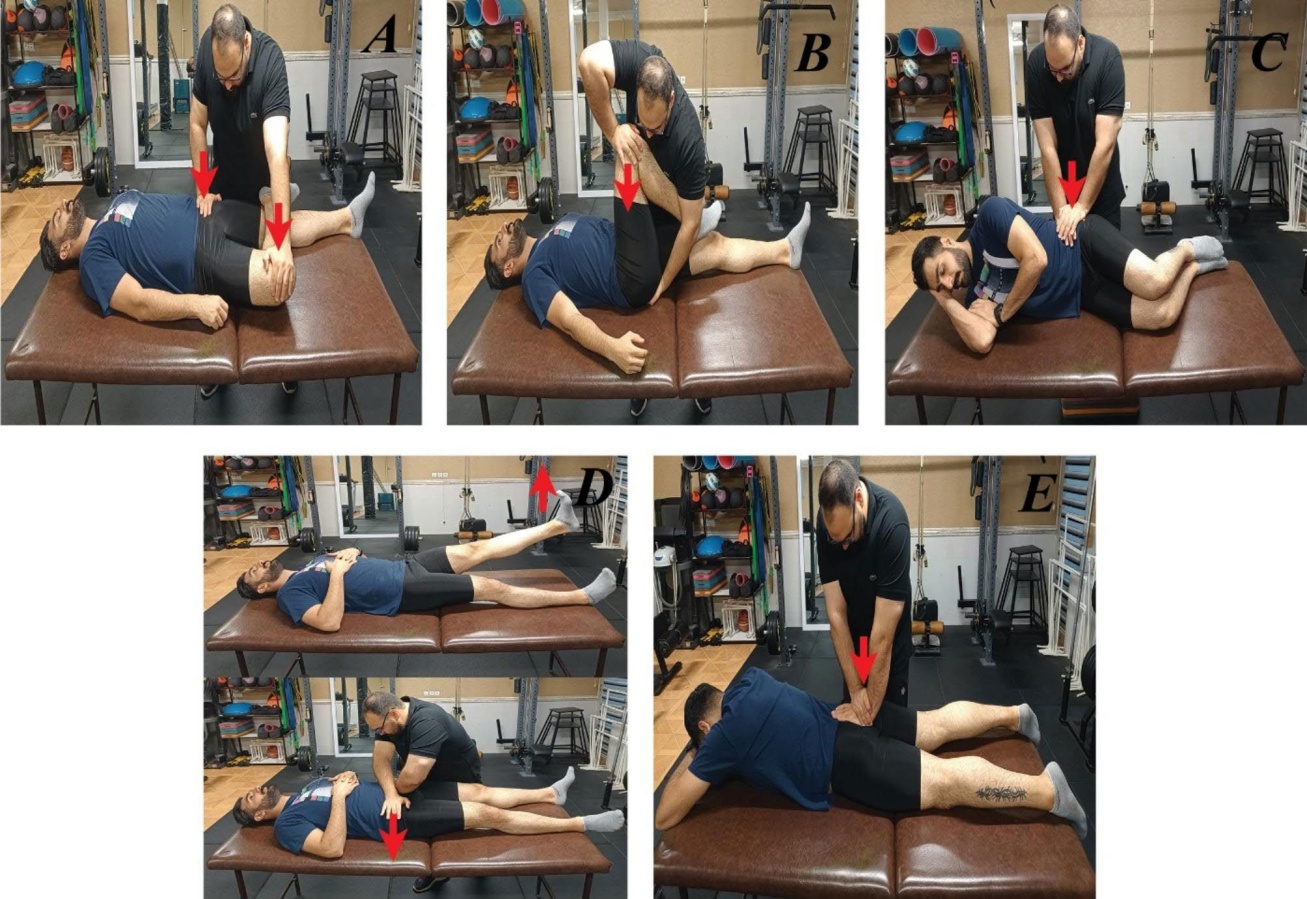
Tests of diagnose SIJ pain, (**A**) the Patrick’s FABER, (**B**) posterior shear, (**C**) sacral compression, (**D**) active SLR, (**E**) sacral thrust tests

**Fig. 2:**
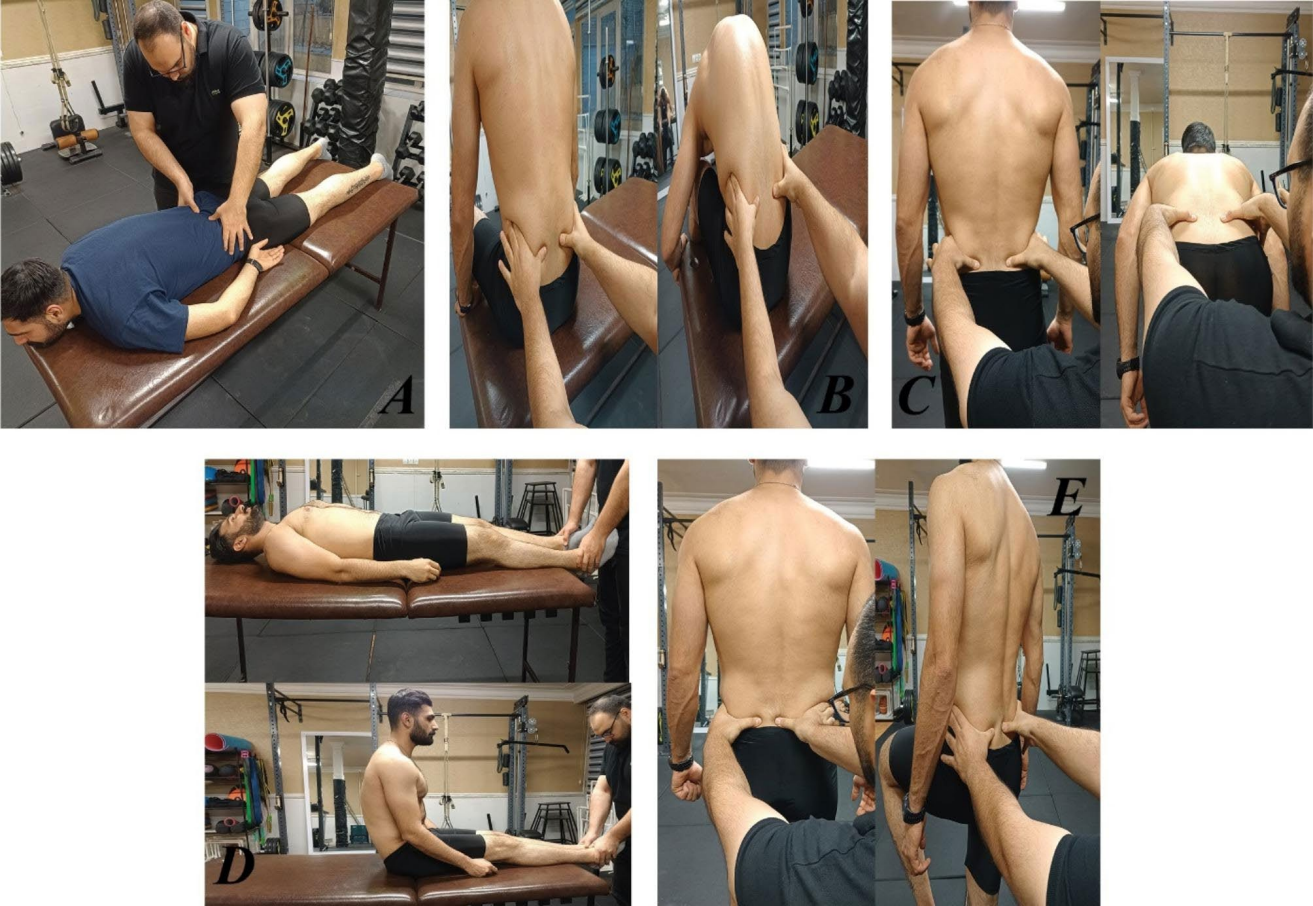
Tests of SIJ movement dysfunction, (**A**) Sacral sulcus, (**B**) sitting flexion, (**C**) Gillet, (**D**) long sitting, (**E**) standing flexion tests

The examiner who was trained in musculoskeletal assessment (MSc on sport injuries and corrective exercises) with three years’ experience completed ten clinical tests in random order (five tests were used to diagnose SIJ pain and a separate set of five tests were used to diagnose SIJ dysfunction). All participants were instructed to wear their own sport clothing during the examination. The specific details of the tests performed are outlined below. This testing sequence was performed one time. On the same day, the participants completed the RIQ. SIJ pain was diagnosed if the patient had a cluster of at least three of five positive provocative tests (Fig. 1). SIJ dysfunction was diagnosed if the patient had a cluster of at least three of five positive clinical tests (Fig. 2). Participants could be categorized as having SIJ pain only, SIJ dysfunction only, both SIJ pain and dysfunction, or no SIJ pain nor dysfunction. While testing, some athletes reported suffering from back pain or regional pain around the SIJ, but the diagnoses of SIJ pain or dysfunction was made only based on the tests results. All tests were performed for both right and left SIJs. A test was considered positive if one of the two sides was positive for pain, therefore the side was not documented.

#### SIJ pain tests

To diagnose SIJ pain, the Patrick’s FABER [29–31], posterior shear [30, 32, 33], sacral compression [34], active SLR [31], and sacral thrust tests were performed. The patient was diagnosed with SIJ pain if 3/5 tests elicited pain during each test. The tests were performed as follows:

Patrick’s FABER test. The subject laid supine on the table with the examiner standing next to him. The examiner brought the ipsilateral hip into flexion, abduction, and external rotation and the knee into flexion so that the heel was on the contralateral knee. Then, the examiner fixed the contralateral anterior superior iliac spine (ASIS) and applied a posteriorly directed pressure on the subject’s flexed knee. The test was positive when buttock or groin pain below L5 was produced [29–31].

##### Posterior shear test

The subject was positioned supine on the table. The examiner flexed the hip and knee so that the hip was approximately in 90 degrees flexion and slight adduction, and the thigh was at the right angle to the table while the knee remained relaxed. While one of the examiner’s hands cupped the sacrum, the other arm and hand wrapped around the flexed knee. An axial pressure applied was directed through the long axis of the femur, which caused anterior to posterior shear to the SIJ. The test was considered positive when pain was provoked over the posterior aspect of SIJ below L5 [30, 32, 33].

##### Sacral compression test

The subject was in a side-lying position, and the examiner’s hands were placed over the upper part of the iliac crest, pressing toward the floor. The movement causes forward pressure on the sacrum. An increased feeling of pressure in the SIJ indicates a possible sacroiliac lesion. A positive result is indicated by pain [34].

##### Active SLR

The subject was lying supine on the table. The examiner stabilized and compressed the pelvis while the patient actively did the straight leg raise providing form closure of the joints by squeezing the innominate bones together anteriorly. If the pain decreased or the SLR was easier to do with form closure (with no increased neurological signs), the test was considered positive for possible SIJ pain [35–37].

##### Sacral thrust test

The subject was lying prone on the table. The examiner applied an anteriorly directed pressure over the sacrum. One hand was placed directly on the sacrum and was being reinforced by the other hand. The purpose is to apply an anterior shear force to both SIJ since the ilia was fixed by the examination bench. The test was positive if the pain was reproduced in the sacroiliac region [31, 34].

#### SIJ dysfunction tests

Sacral sulcus [38–40], sitting flexion [38–40], long sitting [38–40], Gillet [38, 41, 42], and standing flexion [38, 40] tests were used to examine possible SIJ movement dysfunction. These tests were selected for examining the SIJ pain and dysfunction because a combination of motion palpation and provocation tests have been shown to have high reliability for clinical assessment of the SIJ [43].

##### Sacral sulcus test

The subject was lying prone on the table. The examiner palpated the sacral sulcus and inferior angle of the sacrum on each side. The examiner assessed sacral sulci and inferior angles to see if they were symmetrical superiorly / inferiorly [38–40].

##### Sitting flexion test

The procedure was similar to the standing flexion test except that it was performed while sitting on a stool or the edge of treatment table. The test was positive on the side in which the PSIS moves cranially more than the other side, and negative if PSISs move equally [38–40].

##### Long sitting test

The subject was lying down on the table. The examiner grasped the patient’s legs above the ankles to fully flex then extend them (to standardize the position of the legs relative to the pelvis) and then assessed the alignment of the medial malleoli superiorly / inferiorly. The patient was asked to sit up, and the examiner observed whether the superior/inferior alignment of the medial malleoli changed or remained the same. If one medial malleolus moved differently relative to other malleolus, the test finding was considered positive [38–40].

##### Gillet test

While standing, the examiner palpated the posterior superior iliac spine (PSIS). Then, the subject was asked to stand on one leg while pulling the opposite knee up to the chest. The test was repeated with the other leg. With a negative test, the PSIS moves inferiorly during hip and lumbar spine flexion. If the PSIS on the side on which the knee is flexed and pulled to the chest remains at the level of other PSIS or moves down minimally it indicated a positive test [38, 41, 42].

##### Standing flexion test

The standing flexion test was performed by palpating the PSISs while the subject was bending forward from the standing position. The test was negative if PSISs appeared to move equally and symmetrically. It was considered a positive test if one PSIS moved cranially more than the other side [38–40].

### Data analysis

Data were analyzed using SPSS version 24. Descriptive statistics were used to summarize data. The Chi-squared test was done to examine possible correlations between the study variables. In this study, the prevalence of lower extremity and pelvic girdle injuries were considered as independent variables while presence of SIJP and/or SIJD were set as outcome variables. Multivariate logistic regression analysis was run to develop a risk stratification model. Screening performance characteristics of the model were reported (sensitivity, specificity, and odds ratio). Separate regression models were run for overuse and acute injuries. A p-value < 0.05 was considered significant.

## Results

### Characteristics of the participants

A total of 204 male basketball players participated in the study. The demographic data of all participants are summarized in Table 1.

**Table 1:** Demographic characteristics of the participants

Variable	Only SIJP Group (n = 19)		Only SIJD Group (n = 67)		Both SIJP and SIJD (n = 15)		Without SIJD or SIJP Group (n = 103)	
	With injuries (n = 13)	Without injuries (n = 6)	With injuries (n = 53)	Without injuries (n = 14)	With injuries (n = 15)	Without injuries (n = 0)	With injuries (n = 90)	Without injuries (n = 13)
**Age (years)**	25.61 ± 3.22	27.16 ± 6.27	26.58 ± 7.80	26.28 ± 9.91	28.06 ± 9.23	N/A	25.86 ± 7.25	28.53 ± 8.09
**Weight (kg)**	74.00 ± 12.90	72.00 ± 6.98	76.39 ± 9.92	77.92 ± 9.04	78.40 ± 8.32	N/A	76.67 ± 11.21	79.15 ± 17.62
**Height (cm)**	184.69 ± 8.93	177.16 ± 7.02	184.13 ± 6.52	183.00 ± 6.80	186.06 ± 4.93	N/A	183.58 ± 7.71	185.92 ± 13.32
**BMI (kg/m** ^**2**^ **)**	21.78 ± 4.03	23.02 ± 2.94	22.58 ± 2.92	23.27 ± 2.49	22.6 ± 2.58	N/A	22.76 ± 3.02	22.69 ± 2.88
**Age when started to play (years (**	13.32 ± 3.03	12.83 ± 2.22	12.22 ± 2.60	12.85 ± 2.41	11.60 ± 2.44	N/A	12.44 ± 2.78	11.15 ± 2.67
**Training (sessions/week)**	4.23 ± 4.14	2.16 ± 4.02	4.58 ± 4.36	3.78 ± 4.15	4.80 ± 4.36	N/A	4.27 ± 4.02	2.38 ± 2.98
**Training (hours/week)**	6.00 ± 1.41	7.66 ± 0.81	6.49 ± 1.40	6.85 ± 1.29	6.40 ± 1.35	N/A	6.77 ± 1.36	7.53 ± 0.87
**Games/season**	7.46 ± 10.96	5.66 ± 7.84	10.41 ± 10.40	6.78 ± 8.92	9.40 ± 9.32	N/A	9.70 ± 11.96	8.76 ± 9.98

**Notes**: Values are presented as mean ± standard deviation

**Abbreviation**: SIJP: sacroiliac joint pain, SIJD: sacroiliac joint dysfunction, kg: kilograms, cm: centimeters, m: meter, BMI: body mass index.

The results of frequency of SIJ pain and SIJ dysfunction tests are separately presented in Table 2. Overall, 19 participants (9.3%) had only SIJ pain and 67 participants (32.8%) had only SIJ dysfunction, 15 participants (7.3%) had both, and 103 cases (50.4%) had neither.

**Table 2:** Frequency of SIJ pain provocation tests and SIJ dysfunction tests

SIJP pain tests	Results		SIJD dysfunction tests	Results	
	Positive n (%) ^‡^	Negative n (%)		Positive n (%)	Negative n (%)
**FABER test**	9(4.4)	195(95.6)	**Sacral sulcus test**	34(16.7)	170(83.3)
**Posterior shear test**	34(16.7)	170(83.3)	**Sitting flexion test**	82(40.2)	122(59.8)
**Sacral compression test**	34(16.7)	170(83.3)	**Long sitting test**	102(50.0)	102(50.0)
**Active SLR test**	24(11.8)	180(88.2)	**Gillet test**	102(50.0)	102(50.0)
**Sacral thrust test**	34(16.7)	170(83.3)	**Standing flexion test**	82(40.2)	122(59.8)
**Total N (%)** ^**†**^	34 (16.6)	170(83.3)	**Total N (%)**	82(40.2)	122(59.8)

**Notes**: ‡Data are in percentages of total data, the first number is the raw number and the percentage in the parentheses. †subject had been a cluster of at least three positive tests.

**Abbreviation**: SIJP: sacroiliac joint pain, SIJD: sacroiliac joint dysfunction, yes: People with injuries

One hundred seventy-one athletes had sustained LE injuries, of whom 19 (9.31%) had only SIJP, 67 (32.84%) athletes had only SIJD,15 (7.35%) cases had both (SIJP and SIJD), and 103 (50.49%) cases had no SIJD or SIJP. In addition, a total of 464 LE injuries were recorded in all athletes so some athletes had experienced more than one injury during the previous year.

The logistic regression results, by which athletes with SIJ pain and/or dysfunction were identified as the best combination of acute and overuse injury predictors for pelvic girdle and lower extremities, are provided in Table 3. Significant interactions among variables were evident. The logistic regression coefficients for the interaction between SIJ pain and pelvic girdle injuries and lower limb injuries (acute and overuse injuries) were significantly different (p < 0.001). Also, the coefficients for the interaction between SIJ dysfunction and pelvic girdle injuries, and lower limb acute injuries were significantly different (p < 0.001; Table 4).

**Table 3:** Frequency (n (%)), anatomical distribution and injury type by overuse and acute injuries among athletes who reported previous injuries. Some athletes reported more than one injury

Variable	SIJP Group (with injuries = 19)			SIJD Group (with injuries = 67)			Both Group (with injuries = 15)						Without SIJD and SIJP Group (with injuries = 90)		
	Total	Overuse Injuries n (%) ^‡^	Acute Injuries n (%)	Total	Overuse Injuries n (%)	Acute Injuries n (%)	Total	Overuse Injuries n (%)			Acute Injuries n (%)		Total	Overuse Injuries n (%)	Acute Injuries n (%)
**All injuries**	80(100.0)	60(75.0)	20(25.0)	210(100.0)	39(18.5)	171(81.4)	58(100.0)	21(36.2)			37(63.7)		116(100.0)	33(28.4)	83(71.5)
**Injury location**															
**Lower back/pelvis**	28(35.0)	20(33.3)	8(40.0)	52(24.7)	4(10.2)	48(28.0)	9(15.5)		8(38.0)			1(2.7)	4(3.4)	4(12.1)	0(0.0)
**Hip/groin**	0(0.0)	0(0.0)	0(0.0)	5(2.3)	2(5.1)	3(1.7)	4(6.8)		1(4.7)			3(8.1)	3(2.5)	1(3.0)	2(2.4)
**Lower extremities**	4(5.0)	3(5.0)	1(5.0)	6(2.8)	2(5.1)	4(2.3)	3(5.1)		1(4.7)			2(5.4)	4(3.4)	1(3.0)	3(3.6)
**Knee**	15(18.7)	13(21.6)	2(10.0)	45(21.4)	8(20.5)	37(21.6)	10(17.2)		3(14.2)			7(18.9)	21(18.1)	7(21.2)	14(16.8)
**Shin/calf**	13(16.2)	11(18.3)	2(10.0)	16(7.6)	7(17.9)	9(5.2)	9(15.5)		4(19.0)			5(13.5)	13(11.2)	6(18.1)	7(8.4)
**Ankle**	16(20.0)	11(18.3)	5(25.0)	75(35.7)	14(35.8)	61(35.6)	15(25.8)		4(19.0)			11(29.7)	54(46.5)	10(30.3)	44(53.0)
**Foot**	4(5.0)	2(3.3)	2(10.0)	11(5.2)	2(5.1)	9(5.2)	8(13.7)		0(0.0)			8(21.6)	17(14.6)	4(12.1)	13(15.6)
**Injury type**															
**Muscle/tendon**	28(36.3)	20(39.2)	8(30.7)	65(29.1)	19(52.7)	46(24.5)	17(34.6)		8(42.1)	9(30.0)			65(33.1)	22(51.1)	43(28.1)
**Joint/ligament**	27(35.0)	18(35.2)	9(34.6)	101(45.2)	14(38.8)	87(46.5)	16(32.6)		5(26.3)	11(36.6)			93(47.4)	15(34.8)	78(50.9)
**Bone injury**	15(19.4)	11(21.5)	4(15.3)	23(10.3)	0(0.0)	23(12.2)	9(18.3)		5(26.3)	4(13.3)			19(9.6)	4(9.3)	15(9.8)
**Undefined^^**	7(9.0)	2(3.9)	5(19.2)	34(15.2)	3(8.3)	31(16.5)	7(14.2)		1(5.2)	6(20.0)			19(9.6)	2(4.6)	17(11.1)
**Other**	0(0.0)	0(0.0)	0(0.0)	0(0.0)	0(0.0)	0(0.0)	0(0.0)		0(0.0)	0(0.0)			0(0.0)	0(0.0)	0(0.0)

**Table 4:** Diagnostic Values of pain and dysfunction for predicting pelvic girdle injuries, and lower limb injuries

item	SIJP Group(n = 19)				SIJD Group (n = 67)			
	pelvic girdle		lower limb		pelvic girdle		lower limb	
	Overuse injuries	Acute injuries	Overuse injuries	Acute injuries	Overuse injuries	Acute injuries	Overuse injuries	Acute injuries
**Sensitivity (%)**	83.3	16.4	35.6	94.9	83.3	16.4	35.6	94.9
**Specificity (%)**	92.2	97.1	91.0	17.6	92.2	97.1	91.0	17.6
**Odds ratio**	0.017	0.197	0.179	1.583	0.817	0.165	0.739	0.165
**95% Confidence Interval**	0.005–0.056	0.101–0.384	0.082–0.392	0.653–3.840	0.263–2.544	0.070–0.387	0.387–1.411	0.030, -0.184
**P-values**	< 0.001^**^	< 0.001^**^	< 0.001^**^	0.309	0.728	< 0.001**	0.359	< 0.001^**^

Notes: **P-value ,0.05 considered to be statistically significant

## Discussion

The main purpose of this study was to determine the association of SIJ dysfunction and/or pain with a history of lower limb and pelvic girdle injuries in basketball athletes. To do so, we first had to determine the prevalence of SIJP and SIJD in this population. Disorders, like pain and dysfunction in the SIJ, may lead to different changes in the transfer of load through the pelvic girdle [3, 4, 44–47], which may explain the number of injuries reported by athletes with either SIJP or SIJD. In the present sample, 19 (9.3%) cases had SIJP, 67 (32.8%) cases had SIJD, 15 (7.3%) had both (SIJP and SIJD), and 90 (44.1%) cases had no SIJD or SIJP. Thus, over half of our sample had some type of SIJ dysfunction or pain at the time of testing, indicating the potential importance to screen for SIJ problems in basketball athletes. Moreover, the estimated relationship of SIJ pain with a history of overuse and acute pelvic girdle injuries was significant (p < 0.001). Interestingly, SIJD was only associated with history of acute lower extremities and pelvic girdle sport related injuries and no relationship existed between SIJD and overuse injuries. Generally, it seems that the presence of SIJ dysfunction and pain is associated with a history of acute and overuse injuries in the pelvic girdle and lower limb.

A possible mechanism underlying our findings is that a compensatory patterns of muscle activation may occur secondary to pain and/or dysfunction leading to SIJ instability and ultimately, pain [15, 17, 44, 45, 47]. An asymmetry or dysfunction in load transmission could lead to compensatory muscle activation to stabilize the pelvis [15]. The pelvis needs to be stabilized for coordinated movement on the femur for movements that load a single leg [17]. Instability in the pelvic girdle may predispose the tissues to microtrauma injuries while the athletes have to perform high velocity, repetitive and asymmetrical movements during training or matches [23]. The position of the center of gravity is located adjacent to the SIJ [48], making the role of the SIJ critical for load transmission. Thus, SIJ disorders can cause changes in the load transfer in either a distal-to-proximal or proximal-to-distal direction.

Based on our findings, Iranian basketball players with SIJ pain/dysfunction reported more lower extremity and pelvic injuries compared to players without SIJ pain and/or dysfunction. Therefore, it seems that either SIJ pain and dysfunction causes changes in the load transfer of the pelvic girdle to the lower limb which increases risk factors associated with pelvic girdle and lower extremity injuries [17, 18, 20, 49] or changes in mechanics associated with lower extremity or pelvic injuries led to abnormal forces across the SIJ, leading to dysfunction of that joint. The data presented here do not allow for a determination of the direction of causation.

Despite that the prevalence of SIJ problems remained unclear among athletes, it seems that injuries to the SIJ are very common among athletes [50] and there are numerous etiologies for sacroiliac joint injuries [51]. Several studies [7, 8, 13, 52, 53] have shown that a past history of sport related injuries may be associated with an increase in the prevalence of SIJ dysfunction. Like other sport related injuries, injuries to the SIJ often occur as a result of either acute trauma or repetitive microtrauma while performing highly load demanded movements [54]. So, it seems logical that the prevalence of SIJ pain/dysfunction may be high in athletes who are exposed to these traumas. Moreover, our findings suggests that the presence of SIJ pain/dysfunction correlates with a history of lower extremity injuries, although our data do not allow us to determine cause or effect. Regardless, SIJ injuries should get more attention in treatment of lower extremity injuries in athletes due to the high prevalence in our sample and as they may either cause further injuries or be caused by other injuries and should not be ignored.

## Limitations

It is necessary to mention some limitations and biases of our study, including using a retrospective review approach. By using a retrospective approach, we may not have captured SIJP or SIJD when the athletes were also experiencing their other injuries. Additionally, the reliability for various single tests, such as palpation, pain provocation, standing flexion, and other movement tests, has been shown to be poor. To address this issue, a group of tests were used to increase reliability and validity. Also, there may be other perspectives and contributing factors to relationships that were not included in this model. For example, we only included male athletes, so our results cannot be generalized necessarily to female athletes. Due to the nature of the data collection in which athletes recalled any injuries over the last twelve months and then were evaluated for SIJ pain and/or dysfunction, it is impossible to state causation from one type of injury to the other. Moreover, the recorded injuries were self-reported and had not been formally diagnosed by a physician or any other medical provider. Also, in this study we did not record which side had positive SIJ pain or dysfunction as having a positive result on only one side led to a positive result for the test, while the overall injuries in bilateral lower extremities were taken in account. Thus, more evidence is needed to clarify if the side of SIJ pain/dysfunction may be associated with lower extremity injuries on ipsilateral or contralateral sides. Finally, more caution should consider when discussing the generalizability of data. The small odds ratios showed that, even though statistically significant, the relationship between SIJP, SIJD or both with a history of lower extremity or pelvic girdle injuries (acute and chronic) is quite weak.

## Conclusion

The results of this study indicated that SIJ disorders, like pain and dysfunction, are highly prevalent in athletes and are associated with a history of lower limb and pelvic girdle injuries. The presence of SIJ dysfunction and/or pain have been clinically associated with the history of acute and overuse pelvic girdle and lower extremities injuries in athletes. Therefore, it seems that the athletes with SIJ pain or dysfunction may be predisposed to lower extremity injuries. The results of the presents study suggest clinicians should more thoroughly evaluate the SIJ when rehabilitating athletes with lower extremity or pelvic girdle injuries.

## Electronic supplementary material

Below is the link to the electronic supplementary material.

Supplementary Material 1

Supplementary Material 2

## Data Availability

The raw data and material will be available online after publishing the paper as a **supplementary file**1 in the journal. More queries will reply by corresponding author in future.
