# Supplementary material for: The sacroiliac dysfunction and pain is associated with history of lower extremity sport related injuries
Source: BMC Sports Sci Med Rehabil. 2023 Mar 20;15:36. doi: 10.1186/s13102-023-00648-w (PMC10029172; doi:10.1186/s13102-023-00648-w)
Supplement: Supplementary file 2 — Supplementary Material 2 [file 13102_2023_648_MOESM2_ESM.docx]

**Supplementary file 1: Raw data of the study in Microsoft Excel format**
